# Differentially expressed host long intergenic noncoding RNA and mRNA in HIV-1 and HIV-2 infection

**DOI:** 10.1038/s41598-018-20791-6

**Published:** 2018-02-07

**Authors:** Santanu Biswas, Mohan Haleyurgirisetty, Viswanath Ragupathy, Xue Wang, Sherwin Lee, Indira Hewlett, Krishnakumar Devadas

**Affiliations:** Laboratory of Molecular Virology, Division of Emerging and Transfusion Transmitted Diseases, Center for Biologics Evaluation and Research, Food and Drug Administration, 10903 New Hampshire Avenue, Silver Spring, MD 20993-0002 USA

## Abstract

Non-coding RNAs and mRNAs have been implicated in replication, pathogenesis and host response in HIV infection. However, the impact of long intergenic non-coding RNAs (lincRNAs) on HIV-1 and HIV-2 infection is not known. In this study, we have analyzed expression profiles of lincRNAs and mRNAs in monocyte derived macrophages (MDMs) infected with HIV-1/HIV-2 using microarrays. Our study identified many differentially expressed lincRNAs and mRNAs in MDMs infected with HIV-1/HIV-2 compared to uninfected MDMs. Genes involved in glutathione metabolism and lysine degradation were differentially regulated only in HIV-1 infected MDMs. In HIV-2 infected MDMs, CUL 2, SFRS9, and RBBP4 genes were differentially expressed. Furthermore, we found that plasma levels of lincRNA: chr2: 165509129-165519404 and lincRNA: chr12: 57761837-57762303 were better indicators of HIV-1 infection while lincRNA: chr10:128586385-128592960, XLOC_001148 and lincRNA: chr5:87580664-87583451, were better indicators of HIV-2 infection. In summary, our study has demonstrated that there is substantial alteration in lincRNA and mRNA expression in response to HIV-1/HIV-2 infection. These differentially expressed lincRNAs and mRNAs could serve as prognostic and diagnostic biomarkers of HIV infection and help in the identification of new targets for therapy.

## Introduction

The Human Immunodeficiency Virus (HIV) is an RNA virus belonging to the genera of lentiviruses, family of retrovirus that is characterized by its chronic and persistent infection. HIV can be further classified into two major types, HIV-1 and HIV-2. HIV-1 and HIV-2 are characterized by a high degree of genetic variation. HIV-1 can be further divided into major Group M and minor Groups O, N and P. HIV-1 Group M, that is prevalent throughout the world, can be further subdivided based on genetic diversity into eleven different subtypes (A-K). HIV infection ultimately leads to Acquired Immunodeficiency Syndrome (AIDS), which persists as one of the greatest global health challenges of this century^1–3^. Without an effective vaccine in the near future, it is especially imminent and vital to explore and improve the methods of searching for new and novel host biomarkers of infection that could serve as new targets for therapy and aid in prognosis and diagnosis.

HIV-1 and HIV-2 are closely related viruses that share common pathways involved in viral transmission, replication and pathogenesis. Although HIV-1 and HIV-2 share many similar characteristics, major differences exist between them. HIV-1 is more virulent with higher levels of circulating virus than HIV-2. Reports have indicated that HIV-2 infected patients who progress to AIDS live for a longer time and have relatively higher CD4 counts compared to HIV-1 infected patients who progress to AIDS.

A recent study found that many HIV-2 infected patients remain as long term non-progressors and the mean time for progression to AIDS in HIV-2 infected patients was 14.3 years compared to 6.2 years in people infected with HIV-1. The progression time to death was 15.2 years in HIV-2 infected patients and 8.2 years in HIV-1 infected patients^4^. The mechanisms leading to these pathogenic differences are not fully comprehended. Studies to elucidate the pathogenesis and host defense mechanisms involved in HIV-1/HIV-2 infections would help us in future to identify novel diagnostic and therapeutic strategies.

In the whole human genome, only <3% of genomic DNA is transcribed into mRNAs that code for proteins, while >80% of genomic DNA is transcribed into RNAs^5^ that do not encode proteins and are referred to as non-coding RNA (ncRNAs). These non-coding RNA transcripts were previously considered as ‘junk’ in the genome. The ncRNAs are categorized based on their size into small and long non-coding RNAs (lncRNAs > 200 nucleotides in length)^6^. The lncRNAs constitute the bulk of ncRNAs with current databases describing up to ~62 000 long non-coding RNA genes compared to the ~20 000 known protein coding genes^7,8^. LncRNAs can be further categorized as genic (exonic, intronic and overlapping) or intergenic lncRNAs (lincRNAs) according to their location with respect to the nearest protein-coding transcripts^9^. Reports in the literature have indicated that lncRNAs are not cloning artifacts or transcriptional noise but rather important supplements to proteins or crucial regulators functioning in complex networks^10,11^. Studies have demonstrated that non coding RNAs are capable of regulating gene expression during cellular differentiation^12,13^, and governing a wide-repertoire of molecular, biological and genetic processes including transcription^14^, translation^15^, splicing^16^, imprinting^17^, differentiation^18^, chromatin modification^19^, chromatin structure^20^, cell cycle control^21^, cellular structure^22^ and stem cell regulation^23^. In addition, lncRNAs are implicated in performing a wide variety of functions in various pathophysiologic processes and in human diseases^24–27^.

Coding RNAs^28^ and noncoding RNAs, such as microRNAs^29^ and lncRNAs^30^, are involved in the development and progression of HIV-1 infection. We have previously reported that significant variations in amino acid biosynthetic pathways and cytopathic effects occur in HIV-1 and HIV-2 infected PBMCs *in vitro*^28,31^. We also found that the expression of PHGDH and PSAT 1 genes expression were up regulated only in HIV-1 infected PBMCs and SRSF9 gene expression is up regulated only in HIV-2 infected PBMCs^28^. In the current study, we have extended our previous observations to understand the variations of cellular responses and pathogenesis between HIV-1 and HIV-2 infections in MDMs. Macrophages play a key role in the host immune system by phagocytosing and eliminating obligatory pathogens like HIV-1, as well as initiating protective acquired immune responses through antigen presentation to T cells^32^. Macrophages are considered to be the primary long term residence for HIV-1 in the host. Furthermore, HIV-1 is able to evade major antiviral mechanisms of macrophages by exercising a variety of transcriptional process controls or by hijacking cellular machinery and reshaping host gene expression in their favor^6,33^. Studies have shown that among the altered gene expression profiles induced by HIV-1 infection, both lincRNAs and mRNAs are important components. However, the regulation of lincRNAs expression induced by HIV-1 and HIV-2, and the role played by these transcripts in modulating macrophage responses to HIV infection remains unclear. Furthermore, studies elucidating the role of lincRNAs in HIV-1 infection are at a preliminary stage and not much is known about the impact of lincRNAs in HIV pathogenesis, emerging evidence indicates that lincRNAs play an important role in the modulation of host factors and could serve as novel biomarkers and therapeutic targets for many diseases^34,35^.

In this study, we analyzed the gene expression profiles of MDMs infected with HIV-1/HIV-2 using microarrays. Our results showed modulation of the expression of lincRNAs and mRNAs in MDMs infected with HIV-1/HIV-2 compared to uninfected MDMs. GO analysis and KEGG pathway analysis performed on the differentially regulated mRNAs suggested that different pathways and functions are involved in the macrophage response to HIV-1 and HIV-2 infection. Genes involved in glutathione metabolism, lysine degradation and olfactory transduction, were differentially regulated in HIV-1 infected MDMs. Comparatively more genes were differentially regulated in MDMs infected with HIV-2. Notably, genes for proteins like CUL 2, SFRS9 and RBBP4 were differentially regulated in MDMs infected with HIV-2. From our study, two lincRNAs, lincRNA: chr2: 165509129-165519404 and lincRNA: chr12:57761837-57762303, were identified as potential host biomarkers of HIV-1 infection. Similarly, lincRNA: chr10:128586385-128592960, XLOC_001148 and lincRNA: chr5:87580664-87583451, were identified as potential host biomarkers for HIV-2 infection. We also found that lincRNA: chr5:87580664-87583451, lincRNA: chr12:57761837-57762303 and XLOC_001148 could discriminate between HIV-1/HIV-2 infection and may serve as novel host biomarkers that could differentiate between HIV-1/HIV-2 infected patients. In summary, our study has demonstrated that there is substantial alteration in lincRNA and mRNA expression induced by HIV-1/HIV-2 infection, suggesting that these host factors may serve as potential biomarkers of infection and aid in the identification of new targets for therapy, prognosis and diagnosis.

## Results

### Differential regulation of lincRNAs in MDMs infected with HIV-1/HIV-2

**Figure 1:**
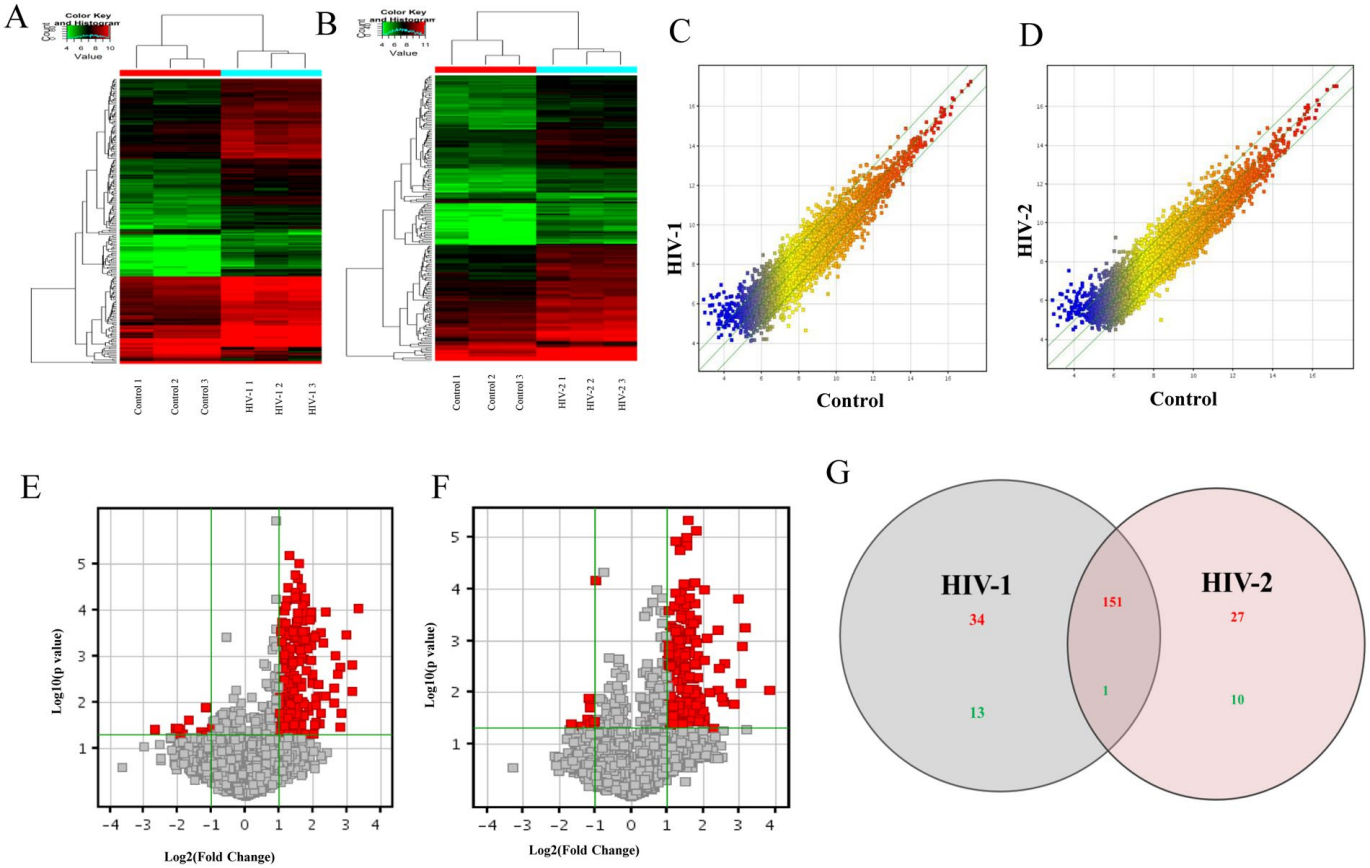
Differential regulation of lincRNAs in HIV-1 infected MDMs, HIV-2 infected MDMs and uninfected MDMs. (**A**) The hierarchical clustering of differentially regulated lincRNAs for HIV-1 and uninfected controls. (**B**) The hierarchical clustering of differentially expressed lincRNAs for HIV-2 and uninfected controls. In the cluster heat map, red indicates high relative expression, and green indicates low relative expression. (**C,D**) Scatter plot of lincRNAs HIV-1 (**C**) or HIV-2 (**D**) infected cells. The values on the X and Y axes of the scatter plot are the normalized signal values of the compared groups (log2 scaled). The green lines are Fold Change Lines (The default fold change value is 2.0). The expression of the genes above the top green line or below the bottom green line indicate greater than 2.0 fold change between the infected and uninfected control groups. (**E,F**) Volcano plots of lincRNA, expression fold change for HIV-1 (**E**) and HIV-2 (**F**) infections in MDMs in comparison to uninfected control: The plot shows differentially regulated lincRNAs with statistical significance that passed Volcano Plot filtering (Fold Change ≥ 2.0 and p-value ≤ 0.05). The vertical green lines correspond to 2.0-fold up and down and the horizontal green line represents a p-value up to 0.05. The red point in the plot represents differentially regulated genes with statistical significance. (**G**) Venn diagrams indicates the numbers of overlapping and non-overlapping differentially regulated lincRNAs in HIV-1 infected MDMs infected and HIV-2 infected MDMs compared to uninfected MDMs Red color indicates upregulation and green color indicates down regulation of lincRNA expression respectively.

To determine the differential regulation profile of lincRNAs in response to HIV-1 and HIV-2 day 7 post infection, microarray analyses were performed on three independent donor MDMs infected with HIV-1/HIV-2. Differentially expressed lincRNAs, with more than two-fold change in expression and with a p value of ≤0.05 were picked for additional study. Hierarchical clustering of the differentially expressed lincRNAs from six samples (Fig. 1A and B) indicated that the differentially expressed lincRNAs were classified into two groups, uninfected control vs HIV-1 infected and uninfected control vs HIV-2 infected. The Scatter plot (1 C &1D) is the visualization method used for evaluating the lincRNAs expression variation (or reproducibility) between arrays. The values of X axes and the Y axes depicted in the Scatter plots represents signal values that are normalized signal values of the groups compared. Green lines represent Fold Change values of the differentially expressed lincRNAs (the default fold change value is 2.0). Figure 1E and F show the volcano plots for the differentially expressed lincRNAs in uninfected control vs HIV-1 infection and uninfected control vs HIV-2 infection respectively.

Data from microarray analysis indicated that 199 lincRNAs (185 upregulated and 14 downregulated) were differentially regulated in HIV-1 infected MDMs and 189 lincRNAs (178 upregulated and 11 downregulated) were differentially regulated in the HIV-2 infected MDMs. Inclusion criteria for upregulated or downregulated lincRNAs were based on at least a 2 fold change in the modulation of their gene expression with a p-value of ≤ 0.05. The complete list of differentially regulated lincRNAs from the HIV-1/HIV-2 infected MDMs compared to uninfected control MDMs is provided in Supplementary Table S1. A total of 151 upregulated lincRNAs (Fig. 1G) and one downregulated (Fig. 1G) lincRNA were found to be common in both HIV-1 and HIV-2 infection. Thirty four lincRNAs (Fig. 1G) and 13 lincRNAs (Fig. 1G) were specifically upregulated and downregulated only in HIV-1 infected MDMs respectively. Similarly, 27 lincRNAs were upregulated (Fig. 1G) and 10 lincRNAs were downregulated (Fig. 1G) only in HIV-2 infected MDMs.

### Differential regulation of mRNAs in MDMs infected with HIV-1/HIV-2

**Figure 2:**
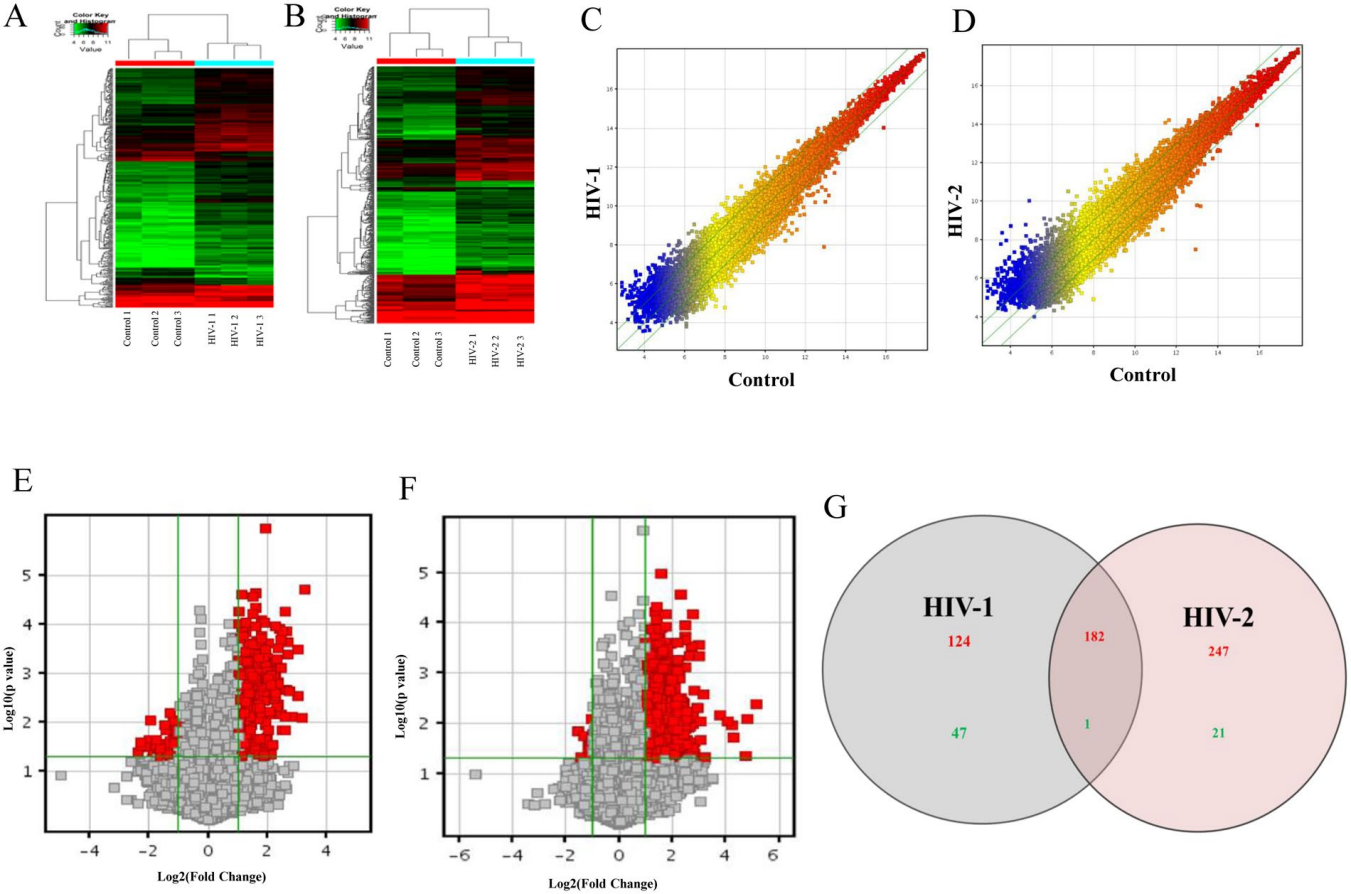
Analysis of differentially regulated mRNAs in MDMs infected with HIV-1, HIV-2 and uninfected controls. (**A**) The hierarchical clustering of differentially regulated mRNAs for HIV-1 and uninfected controls. (**B**) The hierarchical clustering of differentially regulated mRNAs for HIV-2 and uninfected controls. (**C and D**) Scatter plot of mRNAs regulated in HIV-1 (**E**) or HIV-2 (**F**) infected cells. (**E,F**) Volcano plots of the mRNAs, expression fold change for HIV-1 (**E**) and HIV-2 (**F**). The red point in the plot represents the differentially regulated genes with statistical significance. (**G**) Venn diagrams indicates the numbers of overlapping and non-overlapping differentially regulated mRNAs in HIV-1 infected MDMs infected and HIV-2 infected MDMs compared to uninfected MDMs Red color indicates upregulation and green color indicates down regulation of mRNA expression respectively.

Similar to changes in lincRNA regulation pattern, data from microarray analysis identified modulation in the expression of several protein-coding genes that were specific to either HIV-1 or HIV-2 infection. Hierarchical clustering analysis revealed that the differentially expressed genes could readily be classified into two groups, i.e., the HIV-1 vs uninfected control group (Fig. 2A) and HIV-2 vs uninfected control group (Fig. 2B). Figure 2C and D show the Scatter plot for the differentially expressed mRNAs compared to uninfected controls.

From the microarray data, 27,958 coding transcripts were detected, out of which 354 mRNAs approximately 1.26% was differentially regulated in HIV-1 infected MDMs. Among the 354 differentially regulated mRNAs, 306 mRNAs were upregulated and 48 were downregulated (Fold Change ≥ 2 and p value ≤ 0.05) (Fig. 2E). In HIV-2 infected MDMs, 451 out of 27,958 (1.61%) mRNAs showed differential expression, among which 429 mRNAs were upregulated and 22 mRNAs were downregulated (Fold Change ≥ 2 and p value ≤ 0.05) (Fig. 2F). Among the modulated protein-coding genes, 182 upregulated mRNAs (Fig. 2G) and one downregulated mRNA (Fig. 2G) were common in both HIV-1 and HIV-2 infected MDMs. As such, 124 upregulated mRNAs (Fig. 2G) and 47 downregulated mRNAs (Fig. 2G) were specific to HIV-1 infection and 247 upregulated mRNAs (Fig. 2G) and 21 downregulated mRNAs (Fig. 2G) were specific to HIV-2 infected MDMs. The differentially regulated mRNAs in HIV-1/HIV-2 groups are listed in the Supplementary Table S2.

### Functional GO analysis of differentially regulated genes

**Figure 3:**
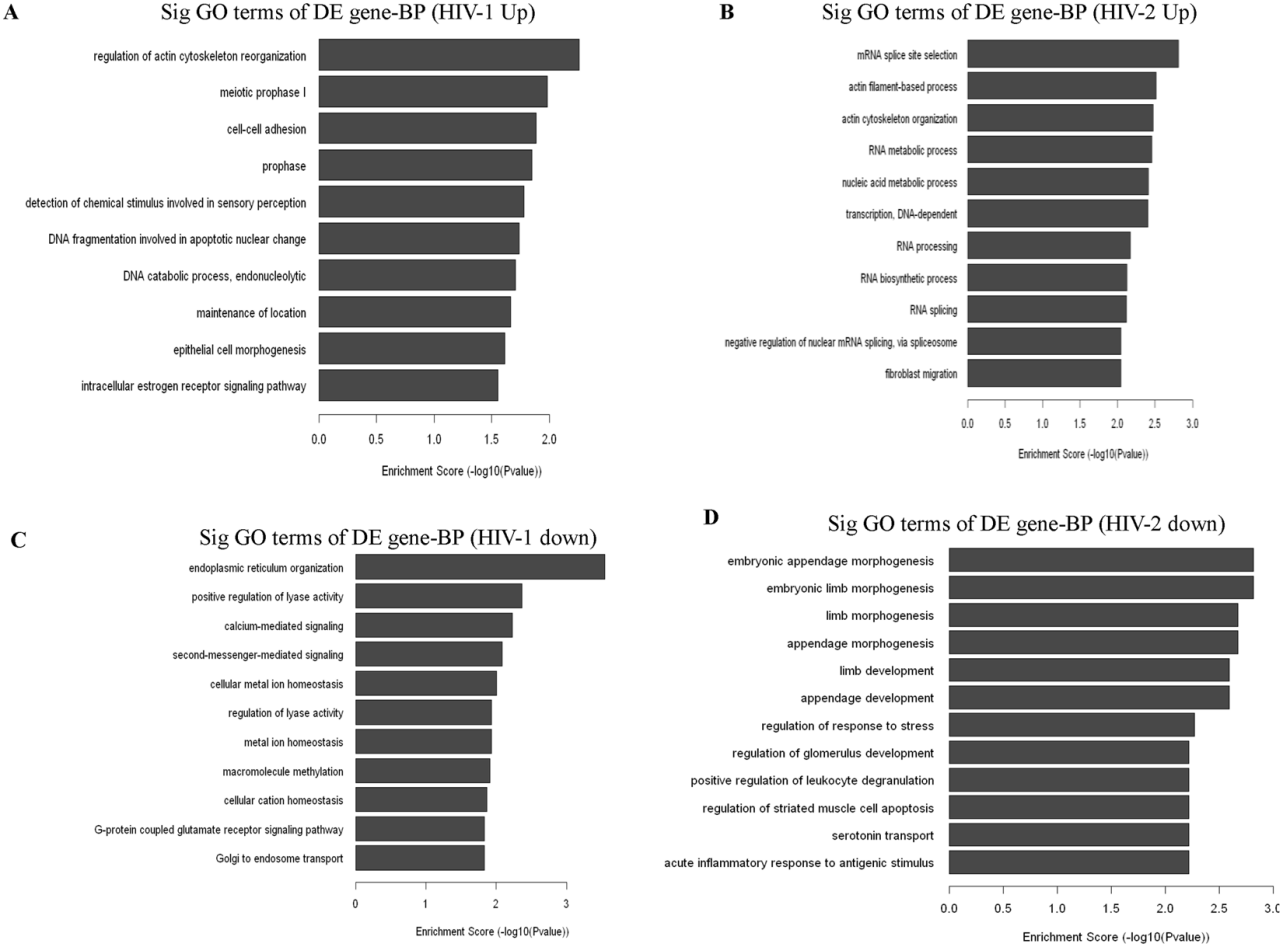
Gene Ontology (biological process) analysis of differentially regulated genes. (**A,C**) The bar plot shows Fold Enrichment value of the significant enrichment terms and pathway analysis for differentially expressed up regulated and down regulated genes in HIV-1 infected MDMs respectively. (**B,D**) The bar plot shows Fold Enrichment value of the significant enrichment terms and pathway analysis for differentially expressed up regulated and down regulated genes in HIV-2 infected MDMs respectively.

Gene enrichment analysis was based on enrichment of the differentially regulated genes. Differentially regulated genes from the microarray analysis were classified into different functional categories based on the biological processes (BP) of the gene ontology classification. The number of significantly enriched GO terms that indicated upregulated mRNAs in HIV-1/HIV-2 infected groups was 26 and 91, respectively. Remarkably, 5 of these GO terms were shared between HIV-1/HIV-2 infected MDMs. In contrast, 48 GO terms (BP) for downregulated mRNAs were enriched only in the HIV-1 infected MDMs and 244 GO terms (BP) for downregulated mRNAs were enriched only in HIV-2 infected MDMs, respectively. Only one GO term was enriched for downregulated mRNAs in both HIV-1/HIV-2 infected MDMs. GO analysis revealed that the regulation of actin cytoskeleton reorganization, cell-cell adhesion, detection of chemical stimulus involved in sensory perception and epithelial cell morphogenesis in (Fig. 3A) were upregulated only in HIV-1 infected MDMs; the downregulated mRNAs were mainly involved in endoplasmic reticulum organization, positive regulation of lyase activity, calcium mediated signaling, second-messenger-mediated signaling, G-protein coupled glutamate receptor signaling pathways and golgi endosome transport (Fig. 3C). Genes involved in mRNA splice site selection, actin filament-based process, actin cytoskeleton organization, RNA processing, RNA splicing and fibroblast migration were found to be up-regulated in HIV-2 infected MDMs (Fig. 3B). The downregulated mRNAs in HIV-2 infected MDMs were mostly related to embryonic appendage morphogenesis, appendage development, regulation of response to stress, serotonin transport and acute inflammatory response to antigenic stimulus (Fig. 3D).

KEGG Pathway analysis was used to investigate the biological pathways of the differentially regulated genes. In HIV-1 infected MDMS, the differentially up regulated genes were mainly related to glutathione metabolism, lysine degradation and olfactory transduction (Supplementary Figure 1). In contrast, differentially up regulated genes in HIV-2 infected MDMs were associated primarily with pathways involved in thyroid cancer, oocyte meiosis, nicotinate metabolism and spliceosome pathways (Supplementary Figure 2). No KEGG pathways were identified to be enriched in the down regulated genes in HIV-1/HIV-2 infected MDMs.

### Validation of microarray results by qPCR and western blot

**Figure 4:**
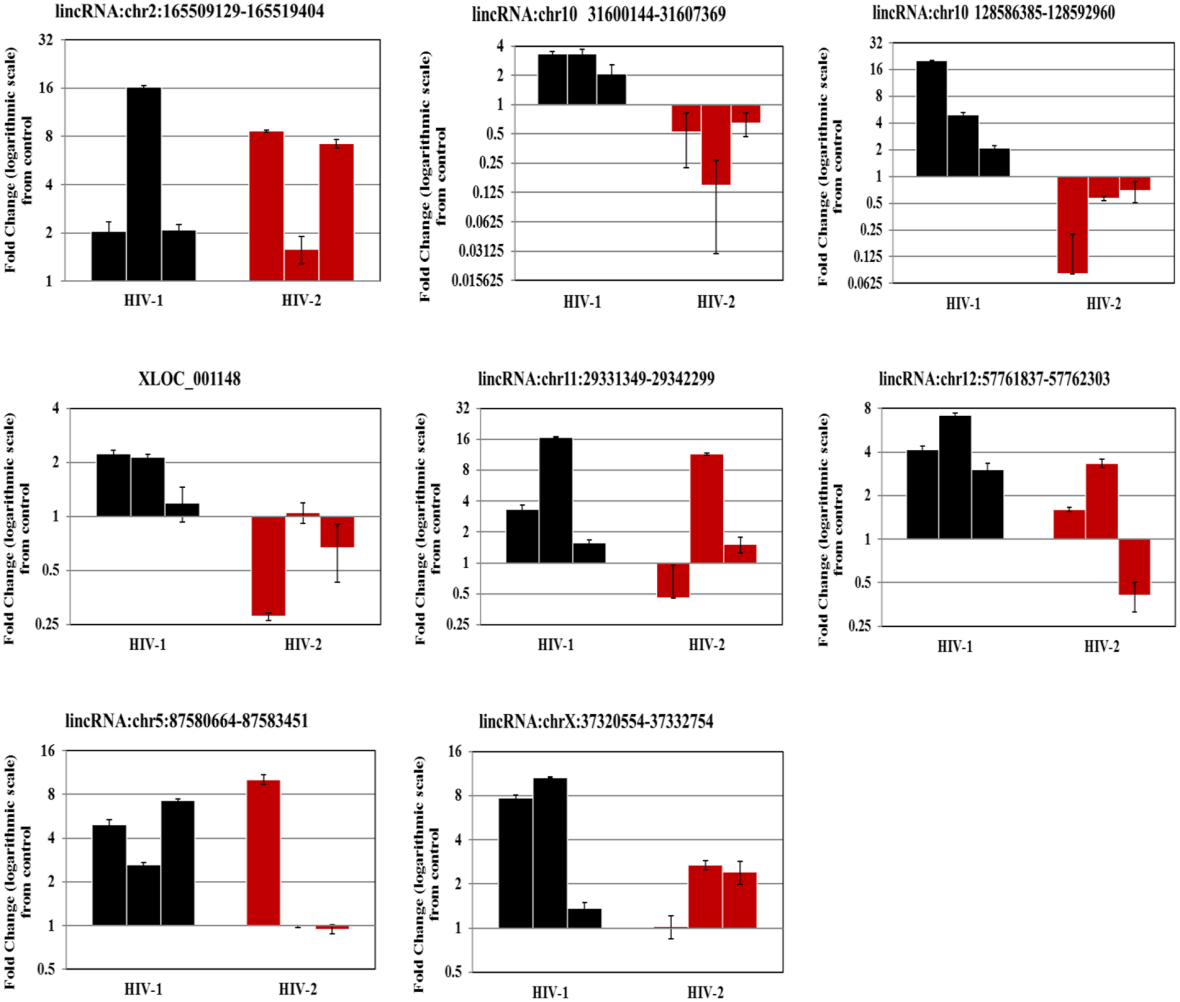
qPCR validation of host lincRNAs in MDMs infected with HIV1 and HIV-2 Day-7 post infection. The data represents results from 3 independent experiments. Values are fold-change plotted on a Log scale for HIV-1 (black) and HIV-2 (red) infected MDMs relative to an uninfected control MDMs. qPCR was normalized with the endogenous GAPDH control and measured the relative amount of target gene. Expression of fold change is calculated as n-fold difference in expression of gene-of-interest relative to GAPDH gene for infected and uninfected cells as n-fold = 2^ − (ΔCt infected −ΔCt uninfected). Each sample was run in triplicate to ensure accurate fold change estimation and the results expressed as mean ± SEM.

**Figure 5:**
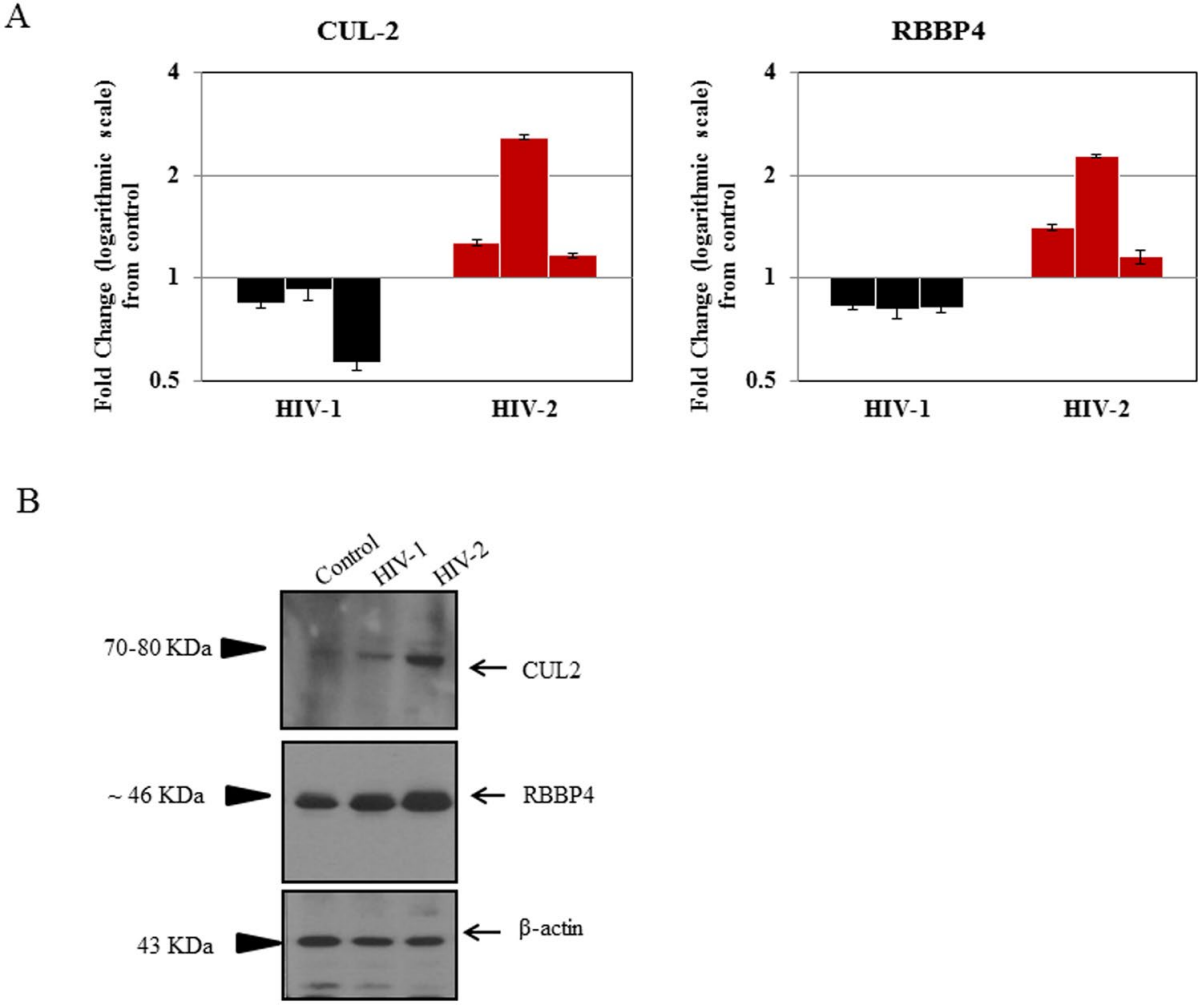
Real-time PCR detection of host genes and western blot detection of host proteins in HIV-1 and HIV-2 infected MDMs Day-7 post infection. (**A**) Real-time PCR data shows results from 3 independent experiments. Values are fold-change plotted on a Log scale for HIV-1 (black) and HIV-2 (red) infected MDMs relative to an uninfected control MDMs. Each qPCR amplification was normalized with endogenous GAPDH control and measured the relative amount of target gene. Expression of fold change is calculated as n-fold difference in expression of gene-of-interest relative to GAPDH gene for infected and uninfected cells as n-fold = 2^ − (ΔCt infected −ΔCt uninfected). Each sample was run in triplicate to ensure accurate fold change estimation and the results expressed as mean ± SEM. (**B**) Western blot data depicts results from proteins isolated from a single donor. Images of the western blots presented here are displayed in cropped format. Full length blots are presented in Supplementary Figure 4. Separate protein gels were run under the same experimental condition and processed in parallel. β-actin was used as a loading control. Western blot data is representative of 3 independent experiments with MDMs derived from 3 independent donors.

To validate our microarray data, 8 lincRNAs were randomly chosen from HIV-1/HIV-2 infected groups (Supplementary Table S1) for detection by quantitative real-time PCR (qPCR). The validation results indicated that 7 lincRNAs had the same pattern of expression in HIV-1 and HIV-2 infected MDMs as that determined by microarray analysis (Fig. 4). One lincRNA; XLOC_001148 showed only a 30% concordance with the microarray results in HIV-2 infected MDMs. The inconsistency between the expression pattern of this lincRNA identified by microarray and by qPCR may be a consequence of donor-to-donor variability that could impact virus infectivity. Similarly, to validate our microarray data (Supplementary Table S2), a subset of differentially regulated genes (Table 1) that demonstrated > 2 fold up or down regulation, coupled with a p-value ≤ 0.05 were selected for detection by qPCR. RT-PCR analysis using day-7 post infection RNA, derived from HIV-1/HIV-2 infected MDMs (Table 1 and Supplementary Figure 3), demonstrates that the RNA samples derived from multiple donors exhibited a consistent gene expression profile which corroborates the microarray results. In HIV-1 infected cells, CRIPI, and MBD5 genes were found to be up regulated (100% agreement with microarray data) and RNF157 and XPNPEP3 found to be down regulated (100% agreement with microarray data) consistent with the gene expression profile detected by microarray analysis. Genes GTF3C3 and FIBIN did not show a consistent pattern of gene expression among the HIV-1 infected MDMs tested. In HIV-2 infected MDMs, genes CEP170, CUL 2, DDX3X, PIKFYVE, RAP2A, RBBP4 and SRSF9 were found to be differentially up regulated, validating the microarray results (Table 1 and Supplementary Figure 3). Protein level changes of some validated differentially expressed mRNAs were determined by western blot. We found that expression of CUL 2 and RBBP4 were up-regulated in HIV-2 infected MDMs compared with uninfected controls (Fig. 5). The western blot results suggest that changes detected in the expression of proteins are consistent with microarray and RT-qPCR data. All validations were done with MDMs isolated from three independent donors that were distinct from MDMs used for the microarray analysis.

**Table 1:** Selected differentially regulated genes in HIV-1 and HIV-2 infected MDMs day-7 post infection.

**Virus**	**Differentially Expressed Gene Symbol**	**Microarray Data Fold change/regulation**	**qPCR Validation % concordance with microarray data**
HIV-1	CRIP1	6.23219/Up	100
HIV-2		5.5059075/Up	100
HIV-1	GTF3C3	4.42145/Up	66.67
HIV-2		8.82112/Up	100
HIV-1	MBD5	6.10735/Up	100
HIV-2		4.245325/Up	66.67
HIV-1	OR5H14	4.95533/Up	100
HIV-2		5.31867/Up	100
HIV-1	RNF157	4.88224/Down	100
HIV-1	XPNPEP3	5.13453/Down	100
HIV-2	CEP170	19.1172/Up	100
HIV-2	CUL2	5.21262/Up	100
HIV-2	DDX3X	27.7645/Up	100
HIV-2	FOS	8.69418/Up	66.67
HIV-2	FURIN	6.81734/Up	100
HIV-2	PIKFYVE	35.2722/Up	100
HIV-2	RAP2A	26.5514/Up	100
HIV-2	RBBP4	7.67802/Up	100
HIV-2	SHPRH	18.3559/Up	100
HIV-2	SNRNP40	2.69952/Up	100
HIV-2	SRSF9	5.70676/Up	100
HIV-2	TRIM52	5.81558/Up	100
HIV-2	ZC3H12D	6.68038/Up	100

### Construction of LincRNA-mRNA coexpression network

A lincRNA-mRNA co-expression network (CNC network) was constructed based on the correlation analysis between the top 50 differentially expressed lincRNAs vs all differentially expressed mRNAs in HIV-1/HIV-2 infected MDMs (Supplementary Tables S3 and S4). Pearson correlation coefficients not less than 0.93 were selected to draw the network using the cytoscape 3.2.1 software. The lincRNA-mRNA co-expression network for HIV-1 infected MDMs identified 237 network nodes associated with 314 edges (Supplementary Figure 5). In HIV-2 infected MDMs, the lincRNA-mRNA co-expression network identified 143 network nodes that were associated with 243 network pairs (Supplementary Figure 6). The results from the CNC network indicated that a single differentially expressed mRNA could correlate with one to ten differentially expressed lincRNAs forming a large correlated network of lincRNAs and host genes.

### Identification of plasma lincRNAs as candidate biomarkers for HIV-1 and HIV-2 infection

**Figure 6:**
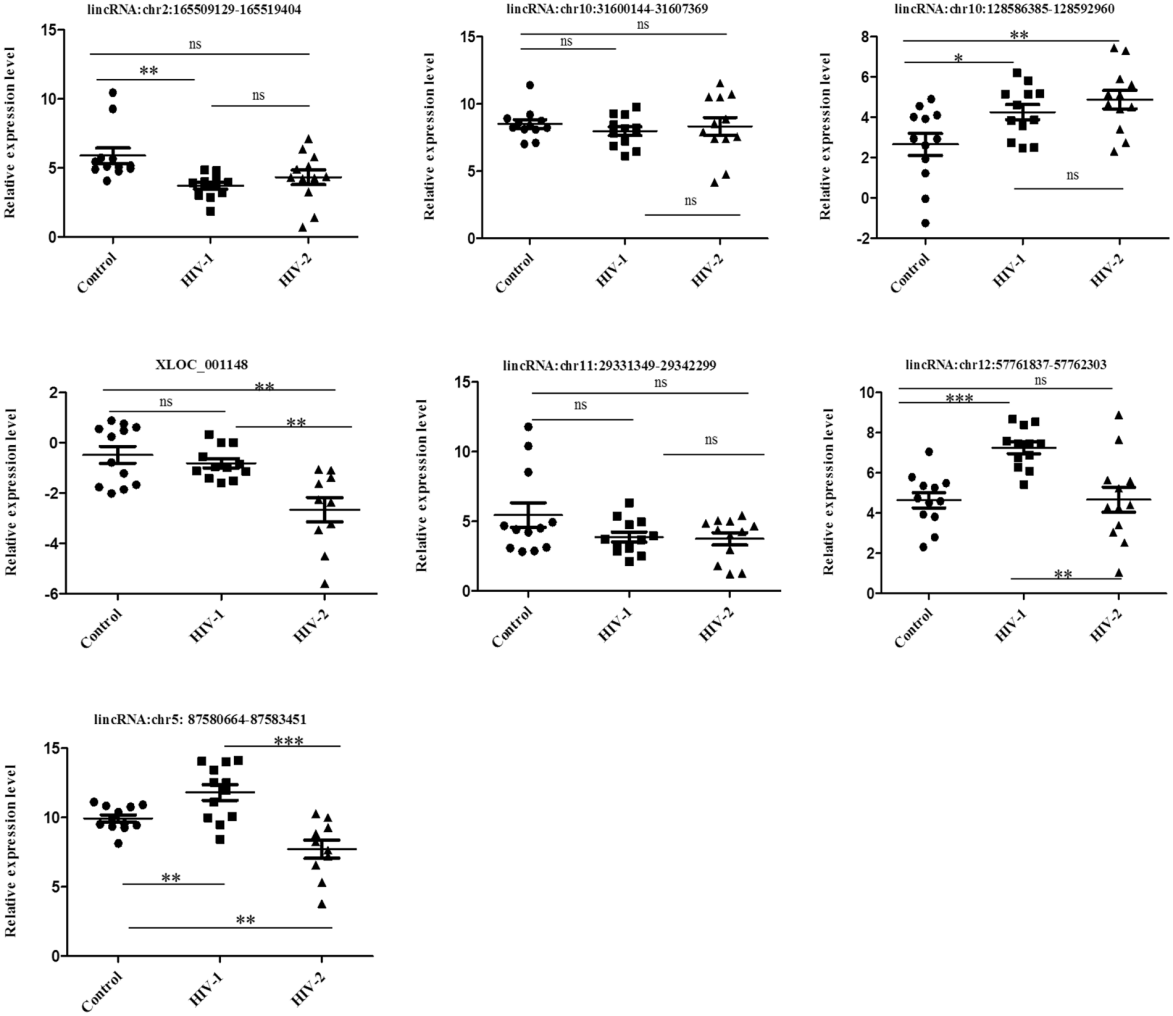
The expression levels of lincRNAs in plasma samples from HIV-1 infected patients, HIV-2 infected patients and healthy controls. Results are expressed as relative expression and the expression was calculated as follows: ΔCt target lincRNA in infected sample = Ct target lincRNA in infected sample - Ct GAPDH in infected sample; ΔCt target lincRNAs in normal = Ct target lincRNA in normal − Ct GAPDH in normal.

**Figure 7:**
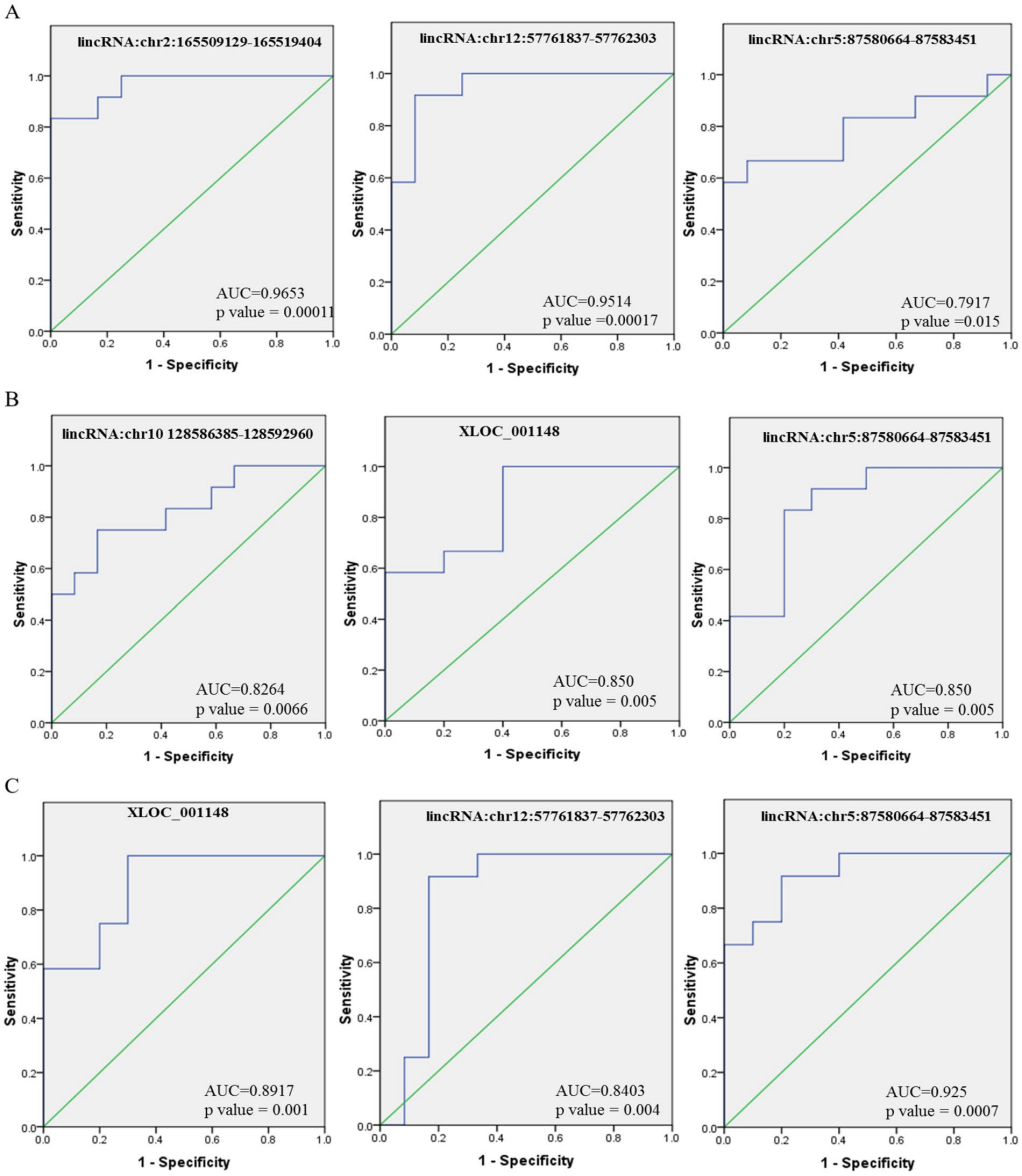
The ROC curve of lincRNAs to evaluate the diagnostic power. (**A**) HIV-1 infected patients vs healthy controls; (**B**) HIV-2 infected patients vs healthy controls; (**C**) HIV-2 vs HIV-1infected patients.

There is an urgent need for the identification of novel and more efficient diagnostic methods based on host biomarkers for a better understanding of HIV-1 and HIV-2 pathogenesis and to devise effective treatment regimens. Recently, many studies have demonstrated that circulatory long noncoding RNA could serve as novel diagnostic markers for a variety of diseases. To determine whether the differentially expressed lincRNAs identified in our microarray analysis could serve as diagnostic biomarkers for HIV-1/HIV-2 infection, expression levels of eight lincRNAs, lincRNA:chr2: 165509129-165519404, lincRNA:chr10: 31600144-31607369, lincRNA:chr10:128586385-128592960, XLOC_001148, lincRNA:chr11:29331349-29342299, lincRNA:chr12:57761837-57762303, lincRNA:chr5:87580664-87583451 and lincRNA:chrX:37320554-37332754 in plasma specimens from HIV-1 and HIV-2 infected patients were compared to specimens from uninfected healthy subjects. One lincRNA (lincRNA: chrX: 37320554-37332754) out of the eight lincRNAs tested, was not amplified and not included for further analysis. The qPCR results showed that compared to healthy controls, the expression of lincRNA: chr2:165509129-165519404 was significantly upregulated in HIV-1 infected patients (p < 0.001) (Fig. 6), while the expression of lincRNA: chr10:128586385-128592960 (p < 0.05), lincRNA: chr12:57761837-57762303 (p < 0.0001) and lincRNA: chr5:87580664-87583451 (p < 0.001) were significantly downregulated in the HIV-1 infected patients (Fig. 6). In HIV-2 infected patients XLOC_001148 (p < 0.001) and lincRNA: chr5: 87580664-87583451 (p < 0.001) lincRNA were significantly elevated and lincRNA: chr10:128586385-128592960 was significantly downregulated (p < 0.001) compared to controls (Fig. 6). No significant differences in the expression of lincRNA: chr10: 31600144-31607369, XLOC_001148 and lincRNA: chr11: 29331349-29342299 were detected in HIV-1 infected patients compared with controls. Similarly, no significant differences in the expression of lincRNA: chr2: 165509129-165519404, lincRNA: chr10: 31600144-31607369, lincRNA: chr11:29331349-29342299 and lincRNA: chr12:57761837-57762303 were detected in HIV-2 infected patients compared with controls. Comparison between HIV-1 and HIV-2 infected patients demonstrated that XLOC_001148 (p < 0.001), lincRNA: chr12:57761837-57762303 (p < 0.001) and lincRNA: chr5: 87580664-87583451 (p < 0.0001) were significantly elevated in only HIV-2 infected patients. Furthermore, to evaluate the power of the 5 dysregulated lincRNAs in detecting HIV-1 and HIV-2 infection, we performed receiver operating characteristic (ROC) curve analysis (Table 2). The results showed that, the area under the curve (AUC) was significantly larger for lincRNA: chr2: 165509129-165519404 (0.9653; 95% CI: 0.9026 to 1.000), lincRNA: chr12:57761837-57762303 (0.9514; 95% CI: 0.8699 to 1.000) and lincRNA: chr5:87580664-87583451 (0.0.7917; 95% CI: 0.6001 to 0.9832) (Fig. 7A) in HIV-1 infected patients compared with healthy controls (Table 2). The AUC for lincRNA: chr10:128586385-128592960 was 0.8264 (95% CI = 0.6603 to 0.9925); the XLOC_001148 AUC was 0.850 (95% CI = 0.6885 to 1.000) and the lincRNA: chr5:87580664-87583451 AUC was 0.850 (95% CI = 0.6824 to 1.000) (Fig. 7B); the AUCs for these lincRNAs were substantially higher than any other lincRNAs in HIV-2 infected patients compared with healthy controls (Table 2). Comparison between the HIV-2 infected patient group with the HIV-1 infected patient group indicated that the AUC was substantially larger for lincRNA: chr5:87580664-87583451 (0.925; 95% CI: 0.8190 to 1.000), lincRNA: chr12:57761837-57762303 (0.8403; 95% CI: 0.6510 to 1.030) and XLOC_001148 (0.8917; 95% CI: 0.7560 to 1.000) (Fig. 7C and Table 2).

**Table 2:** Receiver operating characteristic (ROC) curve analysis of lincRNAs.

**Candidate Biomarkers**	**AUC**	**95% CI**	**Standard error**	**p value**
**ROC curve of lincRNAs to predict HIV-1 patients from controls**				
lincRNAchr2:165509129-165519404	0.9653	0.9026 to 1.000	0.03195	0.000111
lincRNAchr10 128586385-128592960	0.7292	0.5249 to 0.9334	0.1042	0.0568
XLOC_001148	0.5694	0.3082 to 0.8307	0.1333	0.5637
lincRNAchr12:57761837-57762303	0.9514	0.8699 to 1.000	0.04157	0.0001769
lincRNAchr5:87580664-87583451	0.7917	0.6001 to 0.9832	0.0977	0.01535
**ROC curve of lincRNAs to predict HIV-2 patients from controls**				
lincRNAchr2:165509129-165519404	0.7083	0.4870 to 0.9297	0.1129	0.08333
lincRNAchr10 128586385-128592960	0.8264	0.6603 to 0.9925	0.08472	0.00668
XLOC_001148	0.850	0.6885 to 1.011	0.08236	0.005637
lincRNAchr12:57761837-57762303	0.5278	0.2866 to 0.7690	0.123	0.8174
lincRNAchr5:87580664-87583451	0.850	0.6824 to 1.018	0.08549	0.005637
**ROC curve of lincRNAs to predict HIV-2 patients from HIV-1 patients**				
lincRNAchr2:165509129-165519404	0.6944	0.4662 to 0.9227	0.1164	0.106
lincRNAchr10 128586385-128592960	0.5833	0.3486 to 0.8180	0.1197	0.4885
XLOC_001148	0.8917	0.7560 to 1.027	0.06923	0.001952
lincRNAchr12:57761837-57762303	0.8403	0.6510 to 1.030	0.09656	0.004688
lincRNAchr5:87580664-87583451	0.925	0.8190 to 1.031	0.05405	0.0007772

## Discussion

In our study, microarray analysis was used to detect the expression profiles of lincRNAs and mRNAs in three independent donors of MDMs infected with HIV-1/HIV-2 and uninfected controls. Our results identified many differentially expressed lincRNAs and mRNAs in MDMs infected with HIV-1/HIV-2 compared to uninfected cells (Figs 1 and 2). In total, 35% of the differentially regulated lincRNAs were found in HIV-1 infected MDMs and 29% of the differentially expressed lincRNAs were found in HIV-2 infected MDMs respectively, suggesting that these aberrantly expressed lincRNAs play an important role in HIV pathogenesis. Results indicate that more than 100 genes were upregulated in HIV-2 infected MDMs, whereas, in HIV-1 infected MDMs a far greater number of lincRNAs and mRNAs were down-regulated. These results imply that HIV-1 may promote intracellular survival by repressing the expression of genes that aid in viral restriction.

To predict the potential functions of these differentially expressed mRNAs, we carried out GO analysis and KEGG pathway analysis. Analysis demonstrated that most of the enriched GO terms and pathway terms were quite different between HIV-1/HIV-2 infected MDMs. Notably, very few enriched GO terms and pathway terms were shared between HIV-1/HIV-2 infected MDMs. These data suggest that HIV-1 and HIV-2 may evade host defences by using different pathways (Fig. 3). Regulation of actin cytoskeleton reorganization process, identified as an enriched GO term in HIV-1 infected cells, is essential for regulating the expression of HIV-1 induced TNF receptor^36^ which creates an actin specific signaling cascade^37^ that leads to apoptosis of uninfected bystander T cells and to the sustained viral replication in infected macrophages^36^. Interestingly, glutathione metabolism pathway that synthesizes glutathione, also identified as an enriched KEGG pathway in HIV-1 infected cells, plays an important role in antioxidant defense in cells^38^. Altering the glutathione metabolism pathway may lead to glutathione redox in HIV-1 infected macrophages promoting infection^39^. Our microarray and validation results have determined that SNRNP40, SRSF9, TRA2A genes were up regulated in HIV-2 infected MDMs compared to uninfected controls. These genes serve as key regulatory molecules of spliceosome pathways which were also identified as enriched GO terms in HIV-2 infected cells. It has been reported that HIV-1 virus replication was inhibited by promoting excessive splicing of HIV-1 RNA in T cells^40^. HIV-2 may up regulate nuclear mRNA splicing related genes like SRSF9 through the spliceosome pathway to prevent host defense mechanisms. In addition, we found that host genes, CUL 2 and RBBP4 were up regulated in HIV-2 infection only. CUL 2 is a member of the Cullin family and serves as a scaffold protein for Elongin B and C, Rbx1 and various other substrate recognition receptors to form the Cullin-RING E3 ubiquitin ligase complex^41^. In addition, previously we found that CUL-2 was up-regulated in HIV-2 infected jurkat cells^29^. Thus, we speculate that HIV-2 could induce host proteins like CUL 2 which can interact with viral proteins functioning as an E3 ubiquitin ligase to induce the polyubiquitination of host antiviral proteins leading to their degradation. In HIV-1 infected cells, CUL-5, a member of the Cullin family, interacts with HIV-1 protein Vif and functions as an E3 ubiquitin ligase to induce polyubiquitination and proteasomal degradation of host antiviral protein APOBEC3G^42^, thereby promoting HIV-1 infection. RBBP4, also known as NURF55, is a component of several chromatin-related complexes like NuRD and CAF-1^43^. It is known that NuRD complex represses TAT-mediated transactivation of the HIV-1 LTR in T lymphocytes, pointing to a potential role in the initiation of HIV-1 silencing^44^. It is possible that up regulation of RBBP4 protein and the NuRD complex may play a role in the generation of a silenced state of chromatin on the integrated HIV-2 promoter.

The complex modes of action and great abundance make lincRNAs an attractive target to study. Most pathogens like HIV-1 are adept at altering host gene expression profiles in their favor by controlling replication, transcription, and/or translation processes. In fact, few existing reports suggest a role of lncRNAs in activation of HIV-1 replication and subsequent increase in virus production^45–47^. Recently, Nair, *et al*. employed microarray analysis to detect the expression profile of lncRNAs and associated mRNAs in active vs latent HIV-1 infection. The authors revealed that lncRNAs were differentially expressed in U-937 monocytes infected with HIV-1 and suggest that these transcripts may play an important role in regulating the host immune response to HIV-1 infection^48^. Another study by Saayman, *et al*. showed that HIV-1 encoded antisense lncRNAs can epigenetically control HIV-1 transcription^49^. Interestingly, Zhang, *et al*. found that lncRNA SRA was downregulated in both Jurkat and MT4 cells^47^ infected with HIV-1. Studies have indicated that lincRNAs may play a role in apoptosis. LincRNA-p21 is a p53-induced large intergenic noncoding RNA which influences the p53 tumor suppressor pathway by acting in cis as a locus-restricted co-activator for p53-mediated p21 expression. LincRNA-p21 knockdown reduced the effect of p53 mediated apoptosis^50,51^. A recent study suggests that HIV-1 succeeds in inhibiting lincRNA-p21 and the p53 induced apoptotic pathway by activating the MAP2K1/ERK2 pathway, thereby promoting disease progression and viral replication^6^. Other, long noncoding RNAs, NEAT1 and NORN were upregulated in HIV-1 infected cells^46,47,52^. In this study, we discovered that many lincRNAs were differentially regulated in MDMs infected with HIV-1/HIV-2, indicating that lincRNAs might play an important role in the host response to HIV infection.

Based on the CNC analysis of the top 50 lincRNAs and differentially expressed mRNAs in MDMs, we found significant correlations between lincRNAs and multiple protein coding genes. The reported co-expression analysis that predicts interactions between mRNAs and lincRNAs were derived from bioinformatics analysis of the differentially expressed mRNAs and lincRNAs and no direct evidence is presented to substantiate these predictions. Additional gene expression profiling experiments after overexpression or knockdown of specific lincRNAs would be necessary to validate the predicted interactions between lincRNAs and mRNAs. Nevertheless, the CNC analysis data suggests that lincRNAs can impact the transcriptional regulation of mRNA expression both in cis and in trans^53,54^. CNC analysis indicated that many lincRNAs exert their functions through predicted mRNA interactions. For example, we found that lincRNA: chr12:57761837-57762303 was associated with tripartite-motif containing protein 52 (TRIM 52) that has antiviral properties (Supplementary Table S4). TRIM 52, exerts its antiviral activity by degrading viral proteins through their E3 ligase activity or by promoting host innate immunity^55–58^. We also observed a coexistence phenomenon of lincRNA: chr12:57761837-57762303 and SNF2 Histone Linker PHD RING helicase (SHPRH) gene. SHPRH belongs to the RING family of proteins, involved in DNA repair and immunoglobulin diversification^59^. We speculate that lincRNA: chr12:57761837-57762303 may protect host genomic DNA by degrading the viral proteins through the regulation of SHPRH and TRIM 52. Validation results indicated that eight lincRNAs were consistent with the microarray data.

In addition to exploring the function of target genes of the lincRNAs, we expanded the scope of our study to determine whether the lincRNAs were differentially regulated in plasma from HIV-1 infected patients, HIV-2 infected patients, and healthy controls. The detection of circulating nucleic acids in cell-free plasma, serum, and other body fluids^60,61^ has been well documented. Plasma is comparatively easy to obtain and provides new opportunities to develop novel diagnostic or prognostic markers in HIV-1/HIV-2 infection. Several reports have shown that lincRNAs or miRNAs can stably exist in plasma and may be protected by macrovesicles, exosome encapsulation, and apoptotic bodies^62,63^. Therefore, lincRNAs can serve as promising host based biomarkers for diagnosis of diverse HIV variants. In this study, we found 5 lincRNAs, lincRNA: chr2: 165509129-165519404, lincRNA: chr10:128586385-128592960, XLOC_001148, lincRNA: chr12:57761837-57762303 and lincRNA: chr5:87580664-87583451, that were significantly aberrantly expressed in plasma samples from patients with HIV-1/HIV-2 infection in comparison with healthy donors (Fig. 6). We further performed ROC analysis to evaluate the power of these 5 lincRNAs to differentiate between HIV-1 and HIV-2 infected patients from healthy donors as well as to discriminate HIV-2 patients from HIV-1 patients in our cohort. The data indicated that AUC of lincRNA: chr2: 165509129-165519404 and lincRNA: chr12:57761837-57762303 were over 0.9, while that of lincRNA: chr5:87580664-87583451 was lower than 0.8, (Fig. 7) which indicates that lincRNA: chr2: 165509129-165519404 and lincRNA: chr12:57761837-57762303 may function as better candidate host biomarkers for HIV-1 diagnosis. Based on the expression levels as well as AUC of these lincRNAs; lincRNA: chr10:128586385-128592960, XLOC_001148 and lincRNA: chr5:87580664-87583451 might be potential host biomarkers for diagnosis of HIV-2 infection (Table 2). Our data also showed differences in lincRNA: chr5:87580664-87583451, lincRNA: chr12:57761837-57762303 and XLOC_001148 expression signatures between HIV-1/HIV-2 (Table 2), and these 3 lincRNAs could serve as potential diagnostic host biomarkers to differentiate between HIV-1/HIV-2 infected patients. Although, age and sex could have an impact on the expression of the differentially expressed plasma lincRNAs that were identified, confounder effects like age and sex were not taken into consideration during analysis. In this study, our focus was to compare samples from HIV-1/HIV-2 infected patients with samples from un-infected control subjects to identify differentially regulated host factors in response to HIV-1/HIV-2 infection. In future studies, it would be necessary to investigate differentially expressed host factors in larger cohorts factoring other variables like age and sex.

As previously reported, using microarray techniques and subsequent validation by RT-PCR and Western blotting, we identified several host mRNAs that were differentially expressed in PBMCs infected with HIV-1/ HIV-2^28^. In the current report, we have extended our *in vitro* studies to delineate the differential regulation of host mRNAs and long intergenic non-coding RNAs in HIV-1/HIV-2 infected MDMs. Furthermore, in the current study we have identified circulatory lincRNAs in plasma samples from HIV-1/HIV-2 infected patients that may serve as biomarkers of infection. We believe that this is the first report that has comprehensively analyzed and identified both lincRNAs and host mRNAs that are differentially regulated in HIV-1/HIV-2 infected MDMs.

These differentially expressed lincRNAs and mRNAs might be crucial for regulating the antiviral mechanisms of macrophages. More importantly, we have determined that three lincRNAs may have the potential to serve as novel diagnostic host biomarkers for discrimination between HIV-1/HIV-2 infected patient samples. Further validation of these host biomarkers in HIV-1/HIV-2 infection is necessary. We plan to expand this investigation with a larger sample size to verify the identification of the lincRNAs and mRNAs and determine the impact of HIV-1 subtypes on pathogenesis and disease progression.

## Materials and Methods

### Isolation and differentiation of monocytes into monocyte derived macrophages (MDMs)

Human monocytes isolated from PBMC after leukophoresis (buffy coats) of donors seronegative for both HIV-1 and hepatitis B and purified by countercurrent centrifugal elutriation^64^ were provided by the National Institutes of Health (NIH) Blood bank. NIH ethics committee approved this protocol to use deidentified samples of blood and /or blood products originally obtained under the NIH IRB-approved protocol and consent form (study number: 99-CC-0168, PI: Susan F. Leitman, M.D.). The cells were re-suspended in culture medium containing Dulbecco’s Modified Eagle Medium (DMEM) (Quality Biologicals, USA) supplemented with 10% FBS, 100 units/ml Penicillin and 100 µg/ml Streptomycin/ml. After 2 hour of incubation, non-adherent cells were removed and adherent monocytes were cultured for 5–7 days in the presence of 0.02 µg/ml macrophage colony stimulation factor (MCSF, Cat#PHC9504, Thermo Fisher) at density of 10^6^ cells/ml. Cells were judged by morphological examination and found that more than 98% cells were macrophages.

#### Plasma Samples

A total of 24 EDTA plasma samples from HIV-1/HIV-2 infected patients were used for this study. Twelve samples were from HIV-1 infected patients and 12 samples were from HIV-2 infected patients. All samples were purchased from commercial vendors. All plasma specimens were characterized using commercially available FDA-approved/licensed nucleic acid or antibody assays by the vendor. Control plasma samples from donors seronegative for HIV-1, hepatitis B and hepatitis C were obtained from the NIH Blood bank.

### MDMs infection with Viruses

Adherent MDMs were infected with equivalent amounts of 5ng/ml HIV-1 p24 units/million cells of HIV-1 Ba-L (clade B)^65^ (Cat# 510, NIH AIDS Reagent) and 5ng/ml SIV p27 units/million cells of HIV-2 (ROD) (Cat# 0121, NIBSC AIDS Reagent Programme), for 2 hours at 37 °C using previously established protocols^28^.

### Measurement of HIV infectivity in MDMs

Culture supernatants collected from infected MDMs at specified time-points were used to quantitate HIV-1 p24 antigen using NEN/DuPont ELISA analysis kit (Cat# NEK050B00, Perkin Elmer). HIV-2 p26 quantification was done using RETRO-TEK SIV p27 antigen ELISA kit (Cat# 22-156-775, ZeptoMetrix).

### RNA isolation and quality control

Total RNA was extracted as describe earlier [ref] from infected and uninfected MDMs pellets using miRNeasy total RNA isolation kit (cat# 217004, QIAgen) according to the manufacturer’s instructions. Total RNA from each sample was measured using NanoDrop ND-1000. RNA integrity was assessed by standard denaturing agarose gel electrophoresis and Bioanalyser 2100.

### Agilent microarray analysis of lncRNA and mRNA expression

Agilent SurePrint G3 Human Gene Expression 8 × 60 k microarray hybridization and analysis were carried out by ArrayStar Inc. Rockville, MD. This array contains 7,419 lincRNAs probes and 27,958 coding transcript probes, and was constructed using most authoritative public transcriptome database (e.g., RefSeq, Ensemble, Unigene, GenBank, etc.). The RNA samples that passed quality control test were used for the further experiments. The Agilent Array platform was used for the microarray analysis. The sample preparations, microarray hybridization, slide washing and scanning were performed according to the standard protocols as previously described^28^.

### Gene ontology (GO) and pathway analysis

GO analysis was used to investigate biological functions based on differentially expressed coding genes. This analysis classifies functions according to the three following aspects: biological process, cellular component and molecular function. Fisher’s exact test was applied to classify the GO category. The p-value denotes the significance of GO term enrichment in the differentially regulated genes. The lower the p-value signified more the GO term (p value < 0.05). Pathway analysis was used to investigate the differentially expressed coding genes according to the Kyoto Encyclopedia of Genes and Genomes (KEGG). The p-value (EASE-score, Fisher-p value or Hypergeometric-p value) indicates the significance of the pathway correlated with the conditions.  p< 0.05 was considered statistically significant.

### cDNA synthesis and quantitative real-time PCR (qPCR)

RNA samples derived from 3 independent donors were used as templates to validate the microarray results.

Real-time PCR was performed to determine the expression profile of selected mRNAs using Qiagen custom PCR array plates with SYBR-Green and ROX (Cat # 330503 QIAgen) as described by the manufacturer.

Total RNA from uninfected and infected MDMs was reverse transcribed with Superscript III First-Strand Synthesis SuperMix (Cat # 18080400, ThermoFisher). The resulting cDNA was then amplified by using lincRNA specific primers and GAPDH as endogenous control for each donor using SYBR Green chemistry (Cat# A25741 ThermoFisher) as previously discussed^28^. Three independent biological replicates were tested along with three technical replicates for each treatment. Melting curves were analyzed to examine the specificity of amplification. Relative gene expression was calculated by the standard ΔΔCt method.

### Construction of lincRNA-mRNA coexpression network

The lincRNA-mRNA coexpression network was constructed as per Yu *et al*.^66^, using a modified Pearson correlated coefficient (PCC) cut off. The Pearson correlated coefficients were calculated and lincRNA-mRNA pairs were selected that had a Pearson correlated coefficient (PCC) ≥ 0.93 the CNC network was created using cytoscape 3.2.1. Pink nodes represent lincRNAs, blue nodes represent mRNAs. Red solid lines indicate a positive correlation, while the blue equal dashed line indicates a negative correlation.

### Western blot analysis

Validation of mRNA (CUL2 and RBBP4) expression at the protein level was performed by western blot. MDMs infected with HIV-1/HIV-2 and uninfected controls, cultured for 7 days post infection were collected by scrapping followed by centrifugation. Proteins were extracted with RIPA buffer (Cat # 89901, Thermo Scientific) supplemented with protease inhibitor cocktail (Cat # 4693159001, Sigma-Aldrich). Equal amount of proteins (5 µg) were used in the Western blot experiment as described earlier^28^ with m CUL 2 monoclonal antibody (Santa Cruz Biotechnology, Inc., Cat # sc-166506)^67^, RBBP4 monoclonal antibody (Santa Cruz Biotechnology, Inc., Cat # sc-373873)^68^ and β-Actin monoclonal antibody (Santa Cruz Biotechnology, Inc., Cat # sc-47778)^69^. ECL western blotting analysis system (Cat # RPN2108, GE Healthcare) was used to visualize the proteins.

### Statistical Analysis

All results were presented as the mean ± standard error of mean (SEM) of three independent experiments. Differences in lincRNA levels between HIV-1/HIV-2 and control groups were analyzed using unpaired Student t-test. Receiver operating characteristic (ROC) analysis was used to evaluate the power of each candidate biomarker. The statistical analysis was performed using the SPSS 16.0 and GraphPad Prism 5.0 software. All of the statistical tests were two tailed, p values less than 0.05 were considered statistically significant.

## Electronic supplementary material

Supplementary Information

Supplementary Table S1

Supplementary Table S2

Supplementary Table S3

Supplementary Table S4
