# Supplementary material for: Differentially expressed host long intergenic noncoding RNA and mRNA in HIV-1 and HIV-2 infection
Source: Sci Rep. 2018 Feb 7;8:2546. doi: 10.1038/s41598-018-20791-6 (PMC5803214; doi:10.1038/s41598-018-20791-6)

Differentially expressed host long intergenic noncoding RNA and mRNA in HIV-1 and HIV-2 infection

Santanu Biswas, Mohan Haleyurgirisetty, Viswanath Ragupathy, Xue Wang, Sherwin Lee,

Indira Hewlett* and Krishnakumar Devadas*

Supplementary Information

Supplementary Figure 1

Supplementary Figure 2

Supplementary Figure 3

Supplementary Figure 4

Supplementary Figure 5

Supplementary Figure 6

Supplementary Table S1

Supplementary Table S2

Supplementary Table S3

Supplementary Table S4

Supplementary Figure 1: Pathway analyses of up regulated genes in HIV-1 infected MDMs based on latest KEGG (Kyoto Encyclopedia of Genes and Genome database)

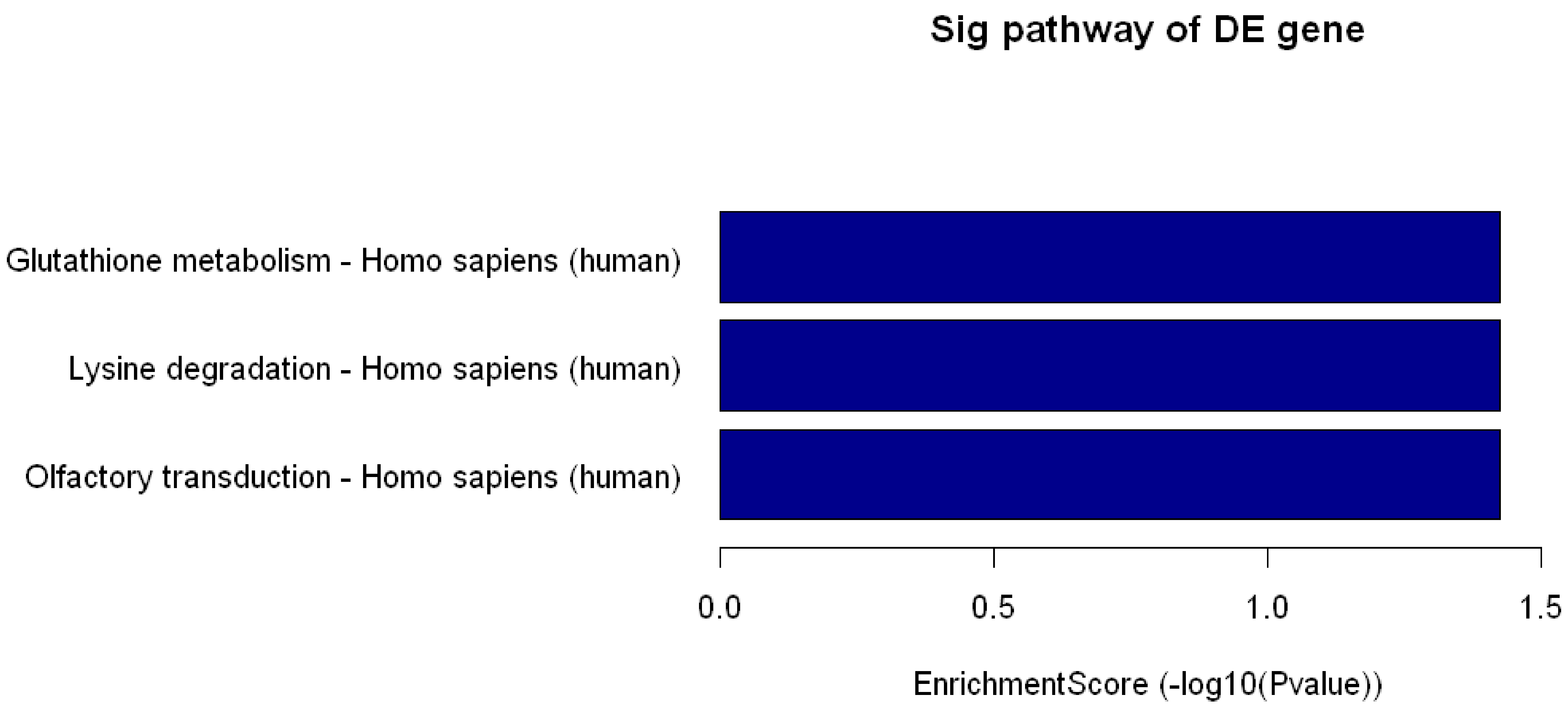

Supplementary Figure 2: Pathway analyses of up regulated genes in HIV-2 infected MDMs based on latest KEGG (Kyoto Encyclopedia of Genes and Genome database)

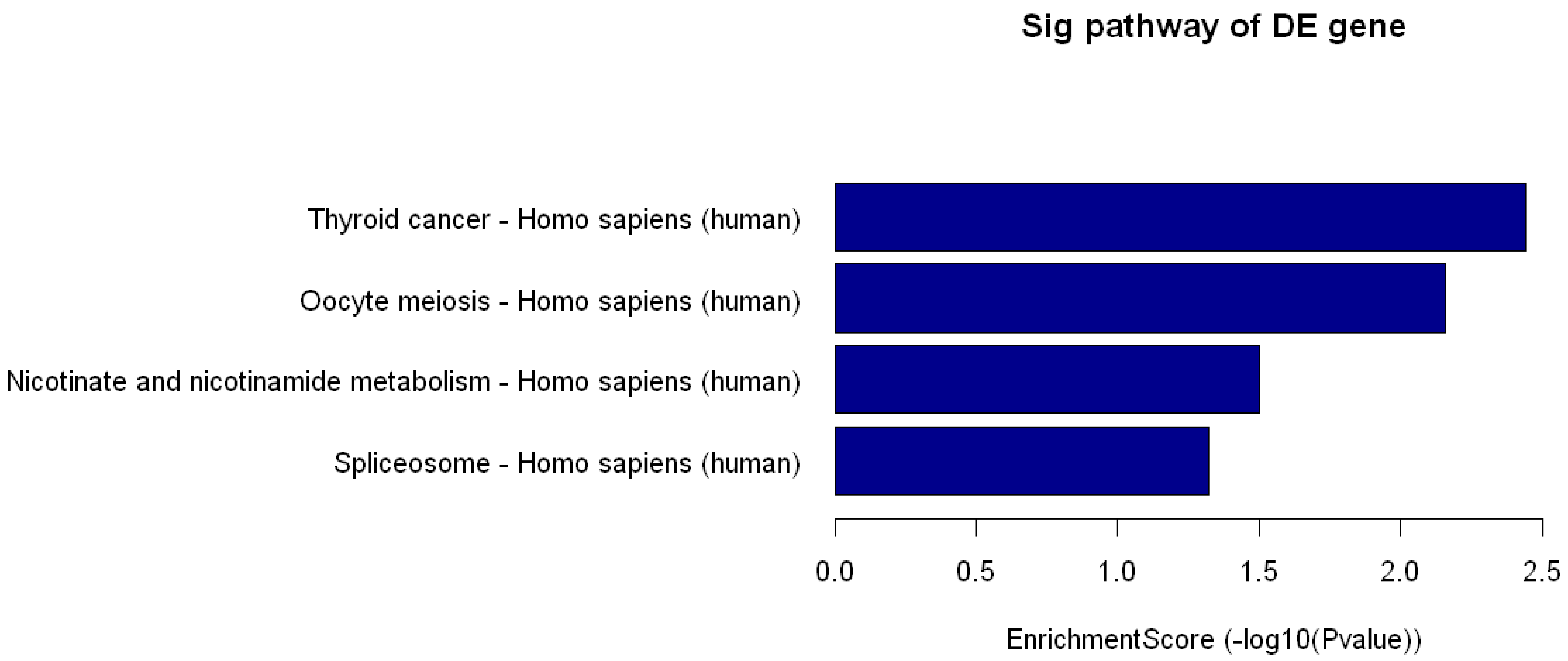

Supplementary Figure 3: qPCR validation of host mRNAs in MDMs infected with HIV-1 and HIV-2 day-7 post infection

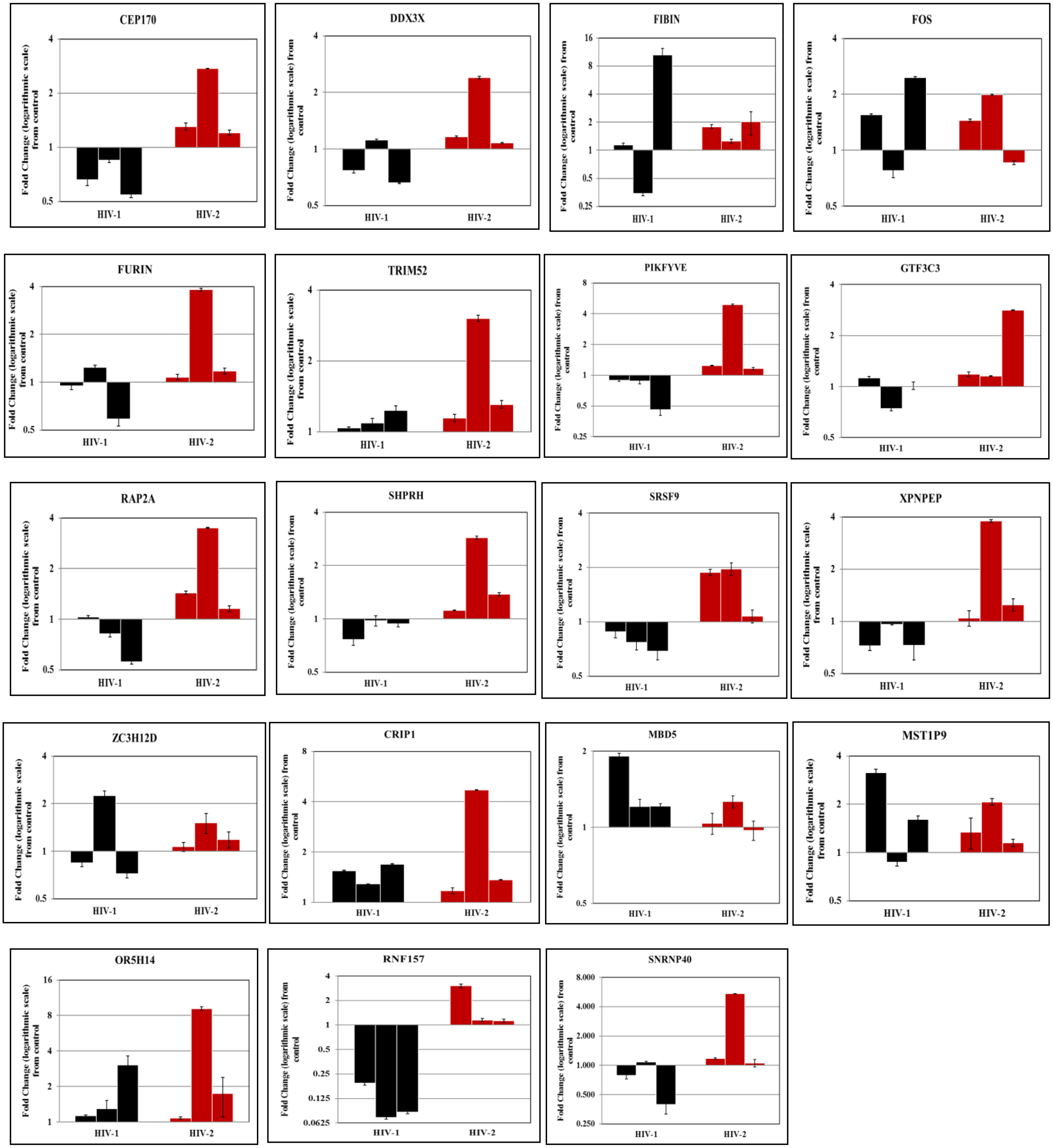

Supplementary Figure 4: Uncropped images of Western Blot for CUL-2, RBBP4 and loading control β-actin shown in Figure 5

CUL-2

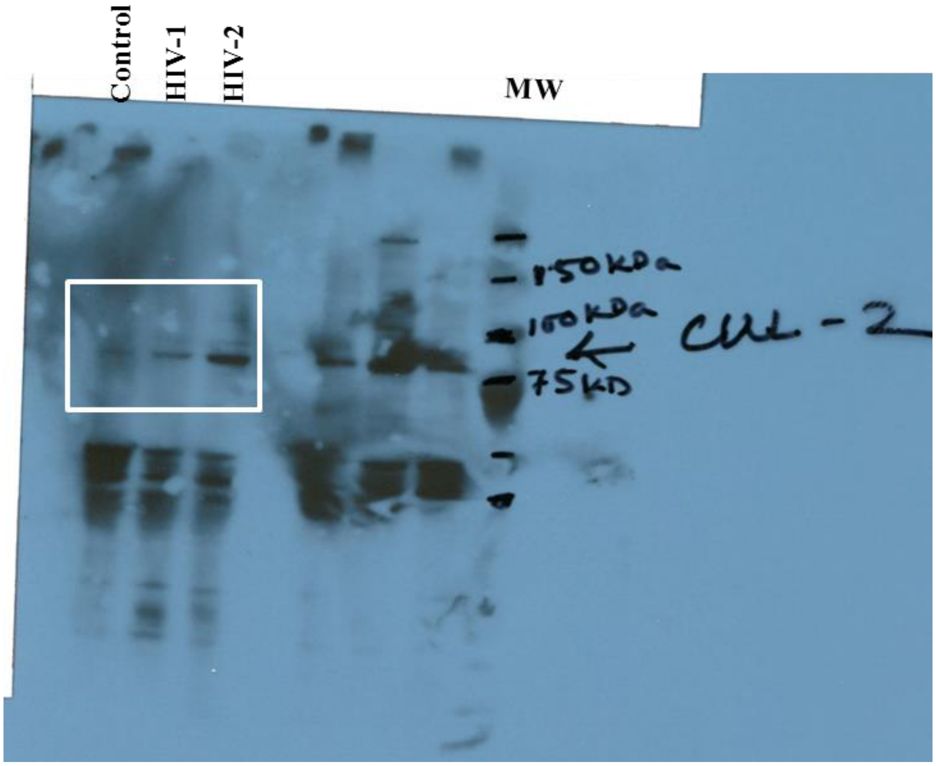

RBBP4 lowexposure

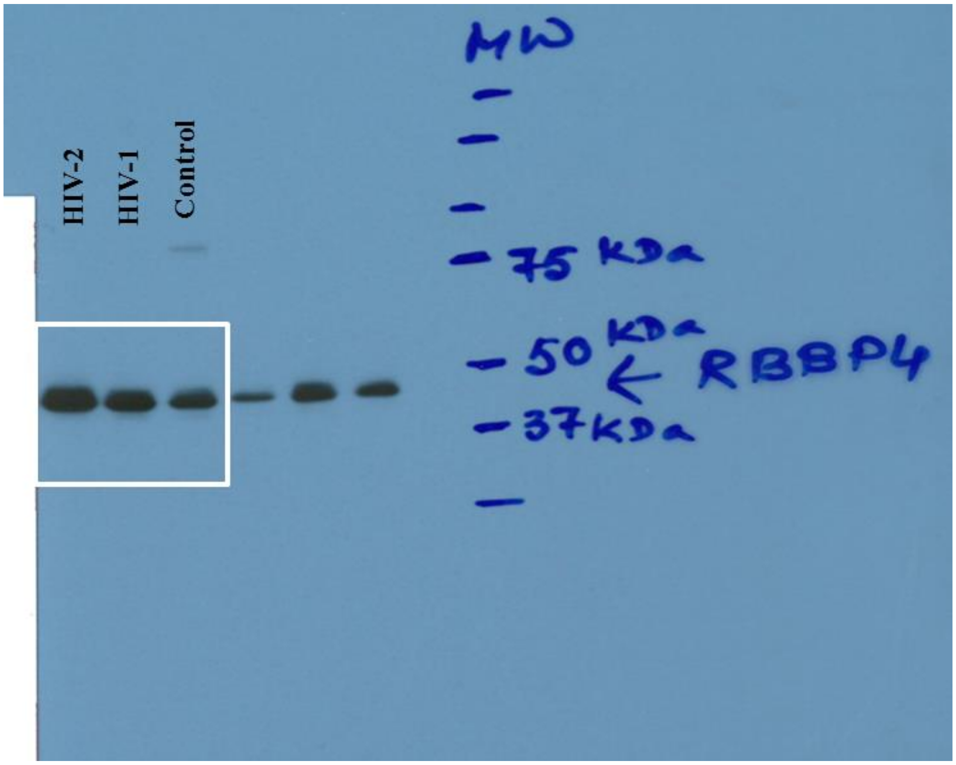

RBBP4 overexposure

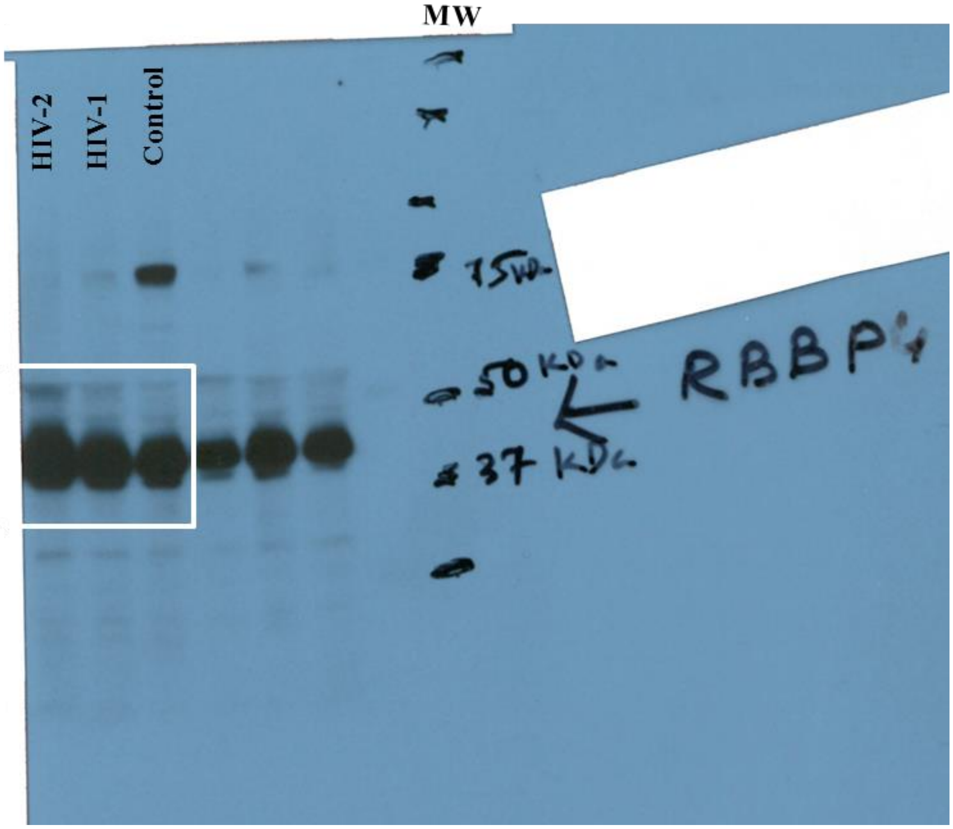

β-actin

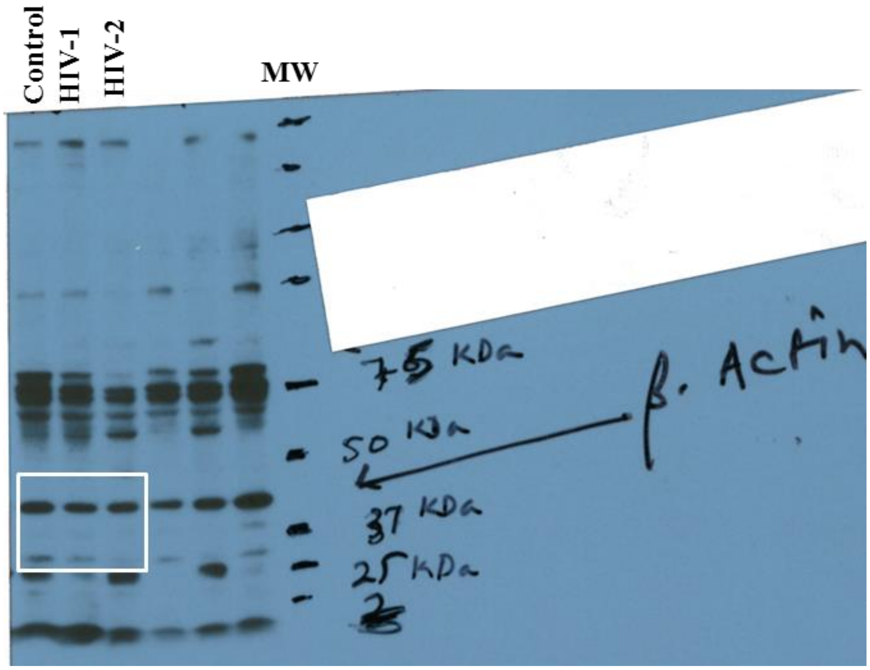

Supplementary Figure 5: HIV-1 lincRNAs-mRNAs coexpression network

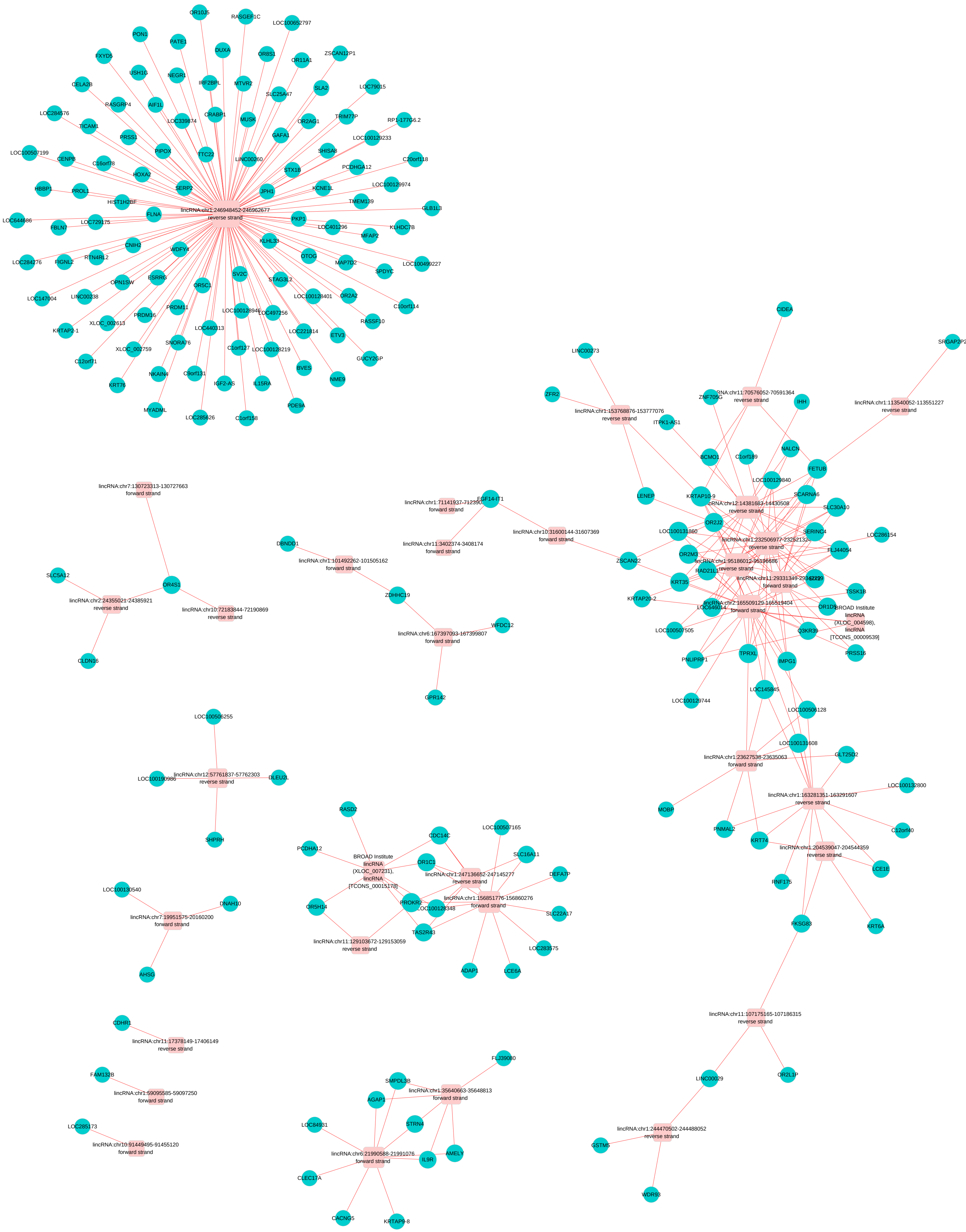

Supplementary Figure 6: HIV-2 lincRNAs-mRNAs coexpression network

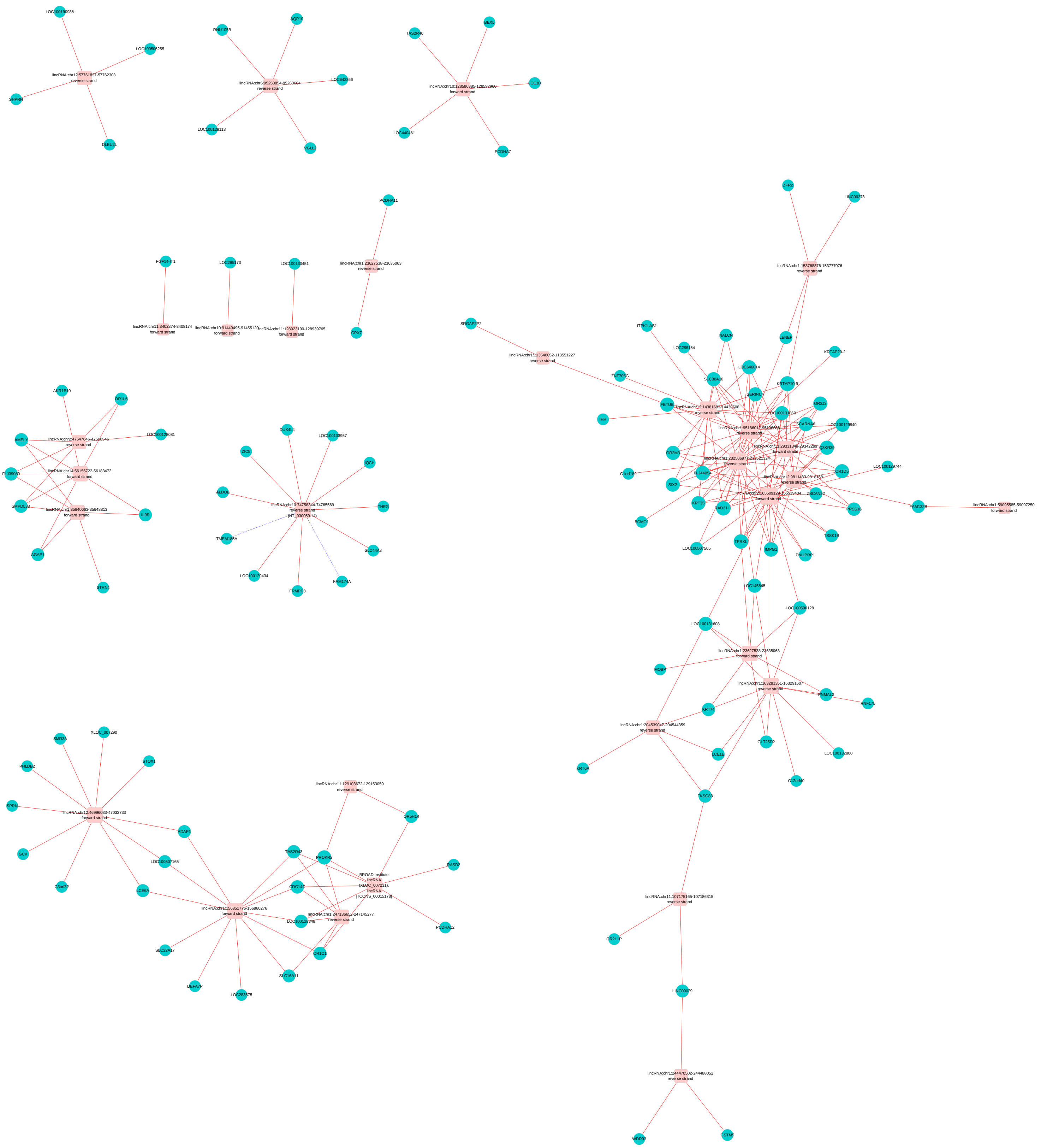

Supplement: Supplementary file 1 — Supplementary Information [file 41598_2018_20791_MOESM1_ESM.pdf]
